# Antibiotic prescribing patterns and consumption at a comprehensive specialized hospital in northern Ethiopia: a prospective cross-sectional study underscoring the need for antimicrobial stewardship

**DOI:** 10.1186/s12879-026-13254-1

**Published:** 2026-04-04

**Authors:** Haftom Yirga Tsegay, Berhane Yohannes Hailu, Gebrehiwot Gebremedhin Tafere, Filmon Beyenne Demoz, Tadelech Yilma Sisay, Muuz Gebru Sahle, Kald Beshir Tuem

**Affiliations:** 1 Department of Veterinary Basics and Diagnostic Sciences, College of Veterinary Sciences, Mekelle University, Mekelle, Ethiopia; 2 Department of Clinical Pharmacy, School of Pharmacy, College of Health Sciences, Mekelle University, Mekelle, Ethiopia; 3 Department of Pharmacology, School of Pharmacy, College of Health Sciences, Mekelle University, Mekelle, Ethiopia; 4 Doctoral School of Biology and Environmental Sciences, University of Debrecen, Debrecen, Hungary

**Keywords:** Antibiotic consumption, Antibiotic resistance, Antimicrobial stewardship, AWaRe classification, Defined daily dose, DU 90%

## Abstract

**Background:**

Antibiotics remain vital for managing bacterial infections; however, their misuse contributes significantly to the rise of antimicrobial resistance (AMR). To guide stewardship and slow resistance, the World Health Organization (WHO) urges regular monitoring of hospital antibiotic use using the Access, Watch, Reserve (AWaRe) analysis, defined daily dose (DDD) metrics, and the drug utilisation 90% (DU90%) index. However, evidence on the patterns of inpatient antibiotic use and consumption in Ethiopia remains limited. This study, therefore, assessed prescribing patterns and consumption at Ayder Comprehensive Specialized Hospital, Northern Ethiopia.

**Methods:**

A prospective cross-sectional study was conducted among inpatients receiving antibiotics in medical, surgical, paediatric, and gynaecology/obstetrics (Gyn/Obs) wards between June and September 2024. Prescriptions were evaluated using the WHO-AWaRe classification, whereas consumption was measured in DDD/100 bed-days and the DU90% index. The data were analysed descriptively.

**Results:**

Among 865 inpatients, 1,491 antibiotic prescriptions were recorded. Ceftriaxone (41%), metronidazole (21.9%), and vancomycin (9.4%) were the most commonly prescribed antibiotics. Watch antibiotics accounted for 64.1% of prescriptions and Access covered 35.9%, deviating from the recommendation that ≥ 70% should be from Access. The total antibiotic consumption was 180.1 DDD/100 bed-days, with ceftriaxone (73.2), metronidazole (45.7), and vancomycin (19.5) being the most frequently consumed antibiotics. The DU90% segment included five antibiotics (ceftriaxone, metronidazole, vancomycin, ceftazidime, and azithromycin), which together accounted for 91.4% of the total consumption.

**Conclusion:**

Antibiotic prescription at this comprehensive specialized tertiary hospital was dominated by Watch antibiotics, particularly ceftriaxone, with overall consumption rates higher than those reported in many regional and global studies. These findings suggest suboptimal antibiotic use in a referral-level setting and emphasize the need for strengthened antimicrobial stewardship programme.

**Clinical trial number:**

Not applicable.

**Supplementary Information:**

The online version contains supplementary material available at 10.1186/s12879-026-13254-1.

## Background

Antimicrobial resistance (AMR) has already become a global threat to health and economies [1]. In 2019, AMR was directly responsible for 1.27 million deaths and was linked to 4.95 million more deaths [2]. By 2021, the toll reached 1.14 million attributable deaths and 4.71 million associated deaths [3]. Projections also warn that by 2050, AMR could cause nearly 2 million attributable deaths and over 8 million associated deaths globally [3].

Although antibiotics play a vital role in managing bacterial infections [1], their misuse and overuse remain key contributors to antibiotic resistance [4, 5]. The World Health Organization (WHO) Global Action Plan on antimicrobial resistance emphasizes the optimization of antimicrobial use and the strengthening of stewardship and surveillance systems worldwide [6]. In alignment with this strategy, Ethiopia has implemented a National Action Plan against AMR to promote rational antimicrobial use, strengthen antimicrobial stewardship [7]. The WHO introduced the Access, Watch, and Reserve (AWaRe) classification as a stewardship tool to guide antibiotic prescribing. While an initial target of at least 60% Access antibiotic use was set under WHO’s 13th General Programme of Work [8], Member States later committed to a more ambitious goal of achieving at least 70% Access use globally by 2030 at the 2024 UN General Assembly High-Level Meeting on AMR [9]. Complementing the AWaRe analysis, the Anatomical Therapeutic Chemical/Defined Daily Dose (ATC/DDD) methodology of the WHO is critical [10]. In relation to this, the drug utilisation 90% (DU90%) index is also a validated method for assessing the quality of antibiotic prescribing [11, 12].

Studies that have assessed antibiotic prescribing patterns via the WHO-AWaRe classification in hospitals are limited in Ethiopia. Reports from Dire Dawa [13] and Adigrat [14] revealed that just over half of prescriptions came from the Access group, whereas Watch antibiotics, particularly ceftriaxone and azithromycin, accounted for the remainder. A study from Jimma Medical Center, southwestern Ethiopia, also indicated that Watch antibiotics accounted for two-thirds of the prescribed antibiotics [15]. Outside Ethiopia, studies in India, Tanzania, and Pakistan similarly revealed imbalances in AWaRe distribution [16–18].

Similarly, antibiotic consumption studies using the DDD/100 bed-day methodology in Ethiopia remain scarce, with one study in Tikur Anbessa Specialized Hospital [19] reporting an average antibiotic consumption of 81.6 DDD/100 bed-days across three wards. Globally, studies using the ATC/DDD methodology have reported varying levels of antibiotic consumption, ranging from 55 to over 90 DDD/100 bed-days, depending on the hospital and clinical setting [20–22]. Additionally, patterns of high cephalosporin consumption and excessive empiric prescription have been documented internationally, including in Sierra Leone [22] and Turkey [20, 21]. A systematic review further confirmed that cephalosporins, penicillins, and fluoroquinolones are the most consumed antibiotic classes globally [23]. With respect to DU90%, the analyses revealed a comparable pattern of concentrated antibiotic use. In Palestine, an orthopedic inpatient study revealed that the consumption of a small number of antibiotics dominated, with ceftriaxone and metronidazole featuring prominently [24]. Similarly, in a study conducted in Indonesia, ceftriaxone was the most common of the five groups of antibiotics associated with DU90% [25].

At Ayder Comprehensive Specialized Hospital (ACSH), previous studies [26–28] have assessed prescribing patterns in specific wards, but there has been no comprehensive evaluation of inpatient antibiotic consumption using the WHO methodologies. In the absence of robust local data, policymakers, stewardship committees, and prescribers lack the necessary evidence to guide the rational use of antibiotics [29]. This creates a risk of unchecked AMR emergence and compromises patient safety. Quantifying inpatient antibiotic consumption using standardized WHO methods can provide essential baseline evidence [8, 10]. Such data are critical for strengthening stewardship programmes, aligning local practices with global targets, and informing future interventions [30]. Therefore, this study aimed to evaluate inpatient antibiotic prescribing patterns and consumption using the WHO-AWaRe classification, DDD/100 bed-days, DU90% index and ACSH, northern Ethiopia.

## Methods

### Study design, study setting and period

A prospective cross-sectional study was carried out among patients receiving antibiotics in the surgical, medical, paediatric, and gynaecology/obstetrics (Gyn/Obs) wards of Ayder Comprehensive Specialized Hospital (ACSH). Situated in Mekelle city, approximately 783 km north of Addis Ababa, ACSH is Ethiopia’s second-largest hospital, with more than 500 inpatient beds and the highest patient load in the region. The hospital serves as both a referral and non-referral centre for more than ten million people across Tigray, Afar, and northern Amhara. Data collection took place between 16 June and 18 September 2024.

### Study population

The study population comprised all patients admitted to the surgical, medical, paediatric, and Gyn/Obs wards of ACSH who fulfilled the eligibility criteria during the study period.

### Eligibility criteria

Patients admitted to the study wards during the study period who received at least one antibiotic were included, while those on long-term programmed therapies, such as antituberculosis or antiretroviral treatment, were excluded.

### Sample size and sampling technique

The sample size (n) was calculated via the single population proportion formula, with no prior study in the setting. With an estimated proportion (P) of 50%, a 95% confidence level (Z = 1.96), and a margin of error (d) of 3.5%, the initial sample size was 785. After adding a 10% contingency to account for potential challenges, the final sample size was set at 865.

$$\:n={\left(Z\right)}^{2}\:P\frac{1-P}{{d}^{2}}$$

**Fig. 1:**
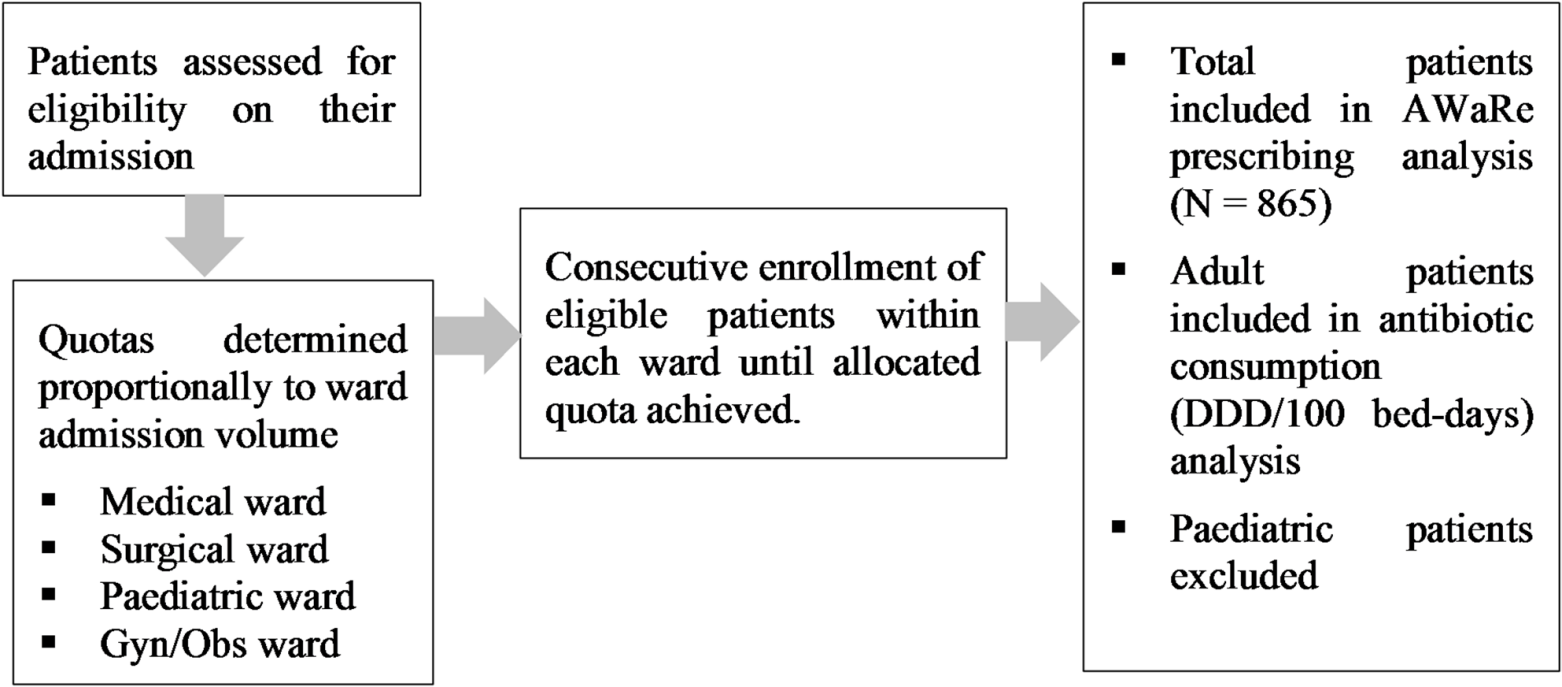
Flow chart illustrating the application of quota sampling technique for selecting inpatients receiving antibiotics across study wards at ACSH

From a total of 1,684 patients admitted to the study wards during the study period, quota sampling was applied by allocating the required sample proportionally to each ward based on patient admission volume. Within each ward, all consecutive inpatient admissions during the study period who met the inclusion criteria were enrolled, until the allocated quota was fulfilled. Any new antibiotic prescribed to the selected patient was recorded as medical chart of patients was reviewed daily. A total of 865 inpatients (adults and paediatrics) were included in the AWaRe antibiotic analysis (Fig. 1). However, as the WHO DDD metric is not validated for paediatric populations [10], only adult inpatients were considered when calculating antibiotic consumption in DDD/100 bed days.

### Data collection tools and techniques

The data collection for this study employed a medical chart review using a structured standard data abstraction format to assess antibiotic prescribing patterns comprehensively via the WHO-AWaRe classification and antibiotic consumption. Antibiotics were categorized according to the WHO’s 2023 AWaRe classification [31]. The ATC codes and DDD information of antibiotics were obtained from the most recent searchable version of the ATC/DDD Index 2025 [32]. The data abstraction format is provided as Appendix A.

### Data quality control and assurance

To maintain data integrity and reliability, several quality assurance measures were implemented. The data abstraction tool was pretested on 5% of the sample (excluded from the main study). Eight trained data collectors, who received comprehensive instruction, conducted the data collection under close supervision. Record completeness was reviewed daily to ensure consistency and accuracy.

### Data management and analysis

The data collected from all the data sources were carefully entered into Microsoft Excel version 2013 and then exported to SPSS version 25 for analysis. An analysis of the sociodemographics of the patients and the WHO-AWaRe classification of antibiotics were performed. Descriptive statistics such as the means ± SDs for continuous variables and frequencies and percentages for categorical variables were computed. For the antibiotic consumption part, the DDD/100 bed days was used and calculated using Microsoft Excel 2013 according to the WHO Collaborating Centre for Drug Statistics Methodology with the following formula:

$$\begin{aligned}&\:\frac{\mathrm{D}\mathrm{D}\mathrm{D}}{100}\mathrm{b}\mathrm{e}\mathrm{d}\:\mathrm{d}\mathrm{a}\mathrm{y}\mathrm{s}\cr&\quad=\frac{\mathrm{T}\mathrm{o}\mathrm{t}\mathrm{a}\mathrm{l}\:\mathrm{c}\mathrm{o}\mathrm{n}\mathrm{s}\mathrm{u}\mathrm{m}\mathrm{e}\mathrm{d}\:\mathrm{u}\mathrm{n}\mathrm{i}\mathrm{t}\mathrm{s}\:\mathrm{i}\mathrm{n}\:\mathrm{g}\mathrm{r}\mathrm{a}\mathrm{m}\times\:100}{\begin{aligned}&\mathrm{D}\mathrm{D}\mathrm{D}\:\mathrm{i}\mathrm{n}\:\mathrm{g}\mathrm{r}\mathrm{a}\mathrm{m}\cr&\times\:\mathrm{b}\mathrm{e}\mathrm{d}\:\mathrm{o}\mathrm{c}\mathrm{c}\mathrm{u}\mathrm{p}\mathrm{a}\mathrm{n}\mathrm{c}\mathrm{y}\:\mathrm{i}\mathrm{n}\mathrm{d}\mathrm{e}\mathrm{x}\cr&\times\:\mathrm{n}\mathrm{u}\mathrm{m}\mathrm{b}\mathrm{e}\mathrm{r}\:\mathrm{o}\mathrm{f}\:\mathrm{b}\mathrm{e}\mathrm{d}\mathrm{s}\cr&\times\:\mathrm{s}\mathrm{t}\mathrm{u}\mathrm{d}\mathrm{y}\:\mathrm{d}\mathrm{u}\mathrm{r}\mathrm{a}\mathrm{t}\mathrm{i}\mathrm{o}\mathrm{n}\:\mathrm{i}\mathrm{n}\:\mathrm{d}\mathrm{a}\mathrm{y}\mathrm{s}\end{aligned}}\end{aligned}$$

The drug DU90% index was determined by ranking antibiotics according to their total consumption in DDD/100 bed-days and identifying the smallest set of agents that together accounted for at least 90% of the overall antibiotic use.

## Results

### Sociodemographic characteristics

Among the 1,684 patients admitted to the study wards, 865 (51.4%) who received at least one antibiotic were included. Slightly more than half were male (50.9%), and the majority came from rural areas (66.2%). The mean age of adults in the medical, surgical, and Gyn/Obs wards was 40 ± 18 years, whereas that of paediatric patients, calculated separately, was 6.3 ± 5.7 years (Table 1).

For a total of 865 inpatients, 1491 prescriptions were made during the study period. In terms of the mean ± SD, patients received 1.7 ± 0.7 antibiotics per patient, and the mean duration of antibiotic therapy was 5.9 ± 3.9 days, where 18.2% of the patients took antibiotics for less than three days, 33.7% for 3–5 days, 27.0% for 6–7 days and 21.1% for greater than seven days. Among the total patients, patients for surgical prophylaxis accounted for the largest share of prescriptions (24.6%), followed by patients with medical conditions (16.6%) and traumatic injuries (12.8%). On the other hand, the most common infections in the study patients were acute appendicitis, pneumonia, nephrotic syndrome, and sepsis of different forms, which were recorded in 7.0%, 6.7%, 5.3% and 4.9% of patients, respectively. A total of 62.4% of the patients had different numbers of comorbidities. Moreover, 21 patients had culture of their samples, of which only 3 had antimicrobial susceptibility tests.

**Table 1:** Sociodemographic characteristics of patients in the study wards, ACSH

Variable	Category	Study wards, *n* (%)				Total *n* (%)
		Surgical	Medical	Paediatric	Gyn/Obs	
Sex	Male	204 (46.4)	117 (26.6)	119 (27.0)	0 (0.0)	440 (50.9)
	Female	70 (16.5)	116 (27.3)	57 (13.4)	182 (42.8)	425 (49.1)
Age (years)	Mean ± SD	36 ± 15	47 ± 20	6.3 ± 5.7	26 ± 8.1	-
Residence	Urban	101 (34.6)	46 (15.7)	84 (28.8)	61 (20.9)	292 (33.8)
	Rural	173 (30.2)	187 (32.6)	92 (16.1)	121 (21.1)	573 (66.2)

### Antibiotic prescribing pattern based on the WHO-AWaRe classification

A total of 17 different antibiotics were prescribed across the study wards, of which nine belonged to the Access class and eight to the Watch class; no Reserve antibiotics were recorded. Among all the prescriptions, 90.9% were injectables. Ceftriaxone was the most frequently prescribed antibiotic (41%), followed by metronidazole (21.9%) and vancomycin (9.4%).

**Fig. 2:**
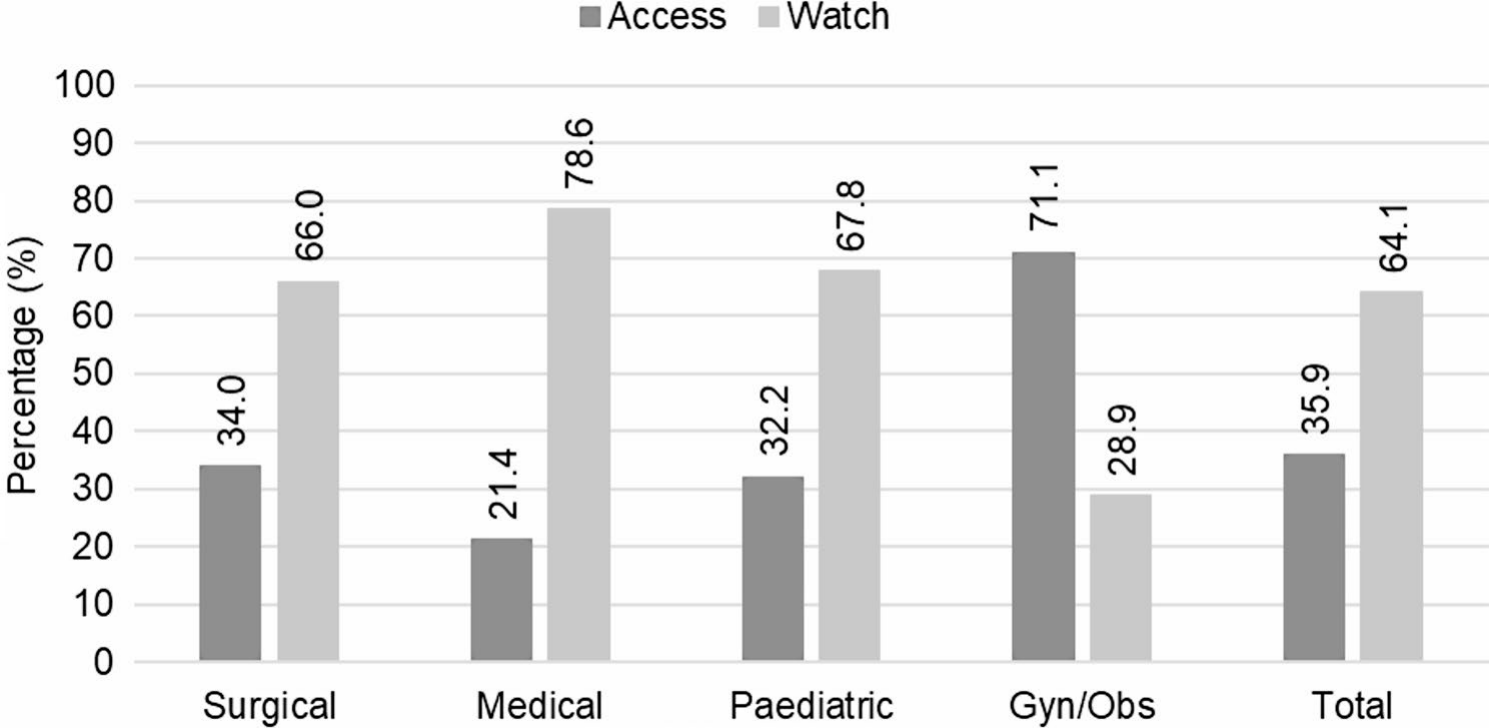
WHO-AWaRe proportions of the prescribed antibiotics in the study wards, ACSH

Nearly two-thirds (64.1%) of the total prescribed antibiotics were from the Watch class of antibiotics, where the medical ward prescribed the highest percentage (78.6%). On the other hand, the total Access antibiotics coverage in the study wards was 35.9%. Based on the study wards, the medical (78.6%), paediatric (67.8%), and surgical (66.0%) wards were found to have more prescriptions of antibiotics from Watch antibiotics than the Access class; whereas the Gyn/Obs ward prescribed a higher percentage of Access antibiotics (71.1%) than the Watch class of antibiotics (Fig. 2).

Metronidazole (61.1%), followed by cefazolin (20.4%), was the most commonly prescribed antibiotic from the Access class, whereas ceftriaxone (64.1%) and vancomycin (14.7%) were the most commonly prescribed antibiotics from the Watch class of antibiotics (Table 2).

**Table 2:** Antibiotic prescribing patterns based on the WHO-AWaRe classification in the study wards, ACSH

Antibiotics	AWaRe Class	Wards, *n* (%)				Total, *n* (%)
		Surgical	Medical	Paediatric	Gyn/Obs	
Gentamycin	Access	0 (0)	4 (0.8)	8 (2.4)	0 (0)	12 (2.2)
Ampicillin	Access	0 (0)	6 (1.3)	31 (9.2)	0 (0)	37 (6.9)
Doxycycline	Access	0 (0)	2 (0.4)	0 (0)	21 (8.3)	23 (4.3)
Cefalexin	Access	1 (0.2)	6 (1.3)	0 (0)	2 (0.8)	9 (1.7)
Metronidazole	Access	146 (33.8)	74 (15.8)	59 (17.4)	48 (19.0)	327 (61.1)
Cefazolin	Access	0 (0)	0 (0)	0 (0)	109 (43.1)	109 (20.4)
Amoxicillin-clavulanic acid	Access	0 (0)	4 (0.8)	0 (0)	0 (0)	4 (0.7)
Trimethoprim-sulfamethoxazole	Access	0 (0)	2 (0.4)	4 (1.2)	0 (0)	6 (1.1)
Amoxicillin	Access	0 (0)	2 (0.4)	7 (2.1)	0 (0)	9 (1.7)
Ceftriaxone	Watch	256 (59.3)	158 (33.8)	134 (39.6)	64 (25.3)	612 (64.1)
Azithromycin	Watch	0 (0)	55 (11.7)	9 (2.7)	2 (0.8)	66 (6.9)
Vancomycin	Watch	14 (3.2)	80 (17.1)	45 (13.3)	1 (0.4)	140 (14.7)
Ciprofloxacin	Watch	1 (0.2)	18 (3.8)	9 (2.7)	0 (0)	28 (2.9)
Cefepime	Watch	0 (0)	4 (0.8)	0 (0)	0 (0)	4 (0.4)
Ceftazidime	Watch	11 (2.5)	43 (9.2)	31 (9.2)	3 (1.2)	88 (9.2)
Cefixime	Watch	0 (0)	0 (0)	0 (0)	2 (0.8)	2 (0.2)
Meropenem	Watch	3 (0.7)	10 (2.1)	1 (0.3)	1 (0.4)	15 (1.6)

### Antibiotic consumption

The overall antibiotic consumption was found to be 180.1 DDD/100 bed-days. The highest DDD/100 bed-days was observed for ceftriaxone (73.2 DDD/100 bed-days), followed by metronidazole (45.7 DDD/100 bed-days) and vancomycin (19.5 DDD/100 bed-days). The lowest DDD/100 bed-days was observed for cefixime (0.07 DDD/100 bed-days) (Table 3).

**Table 3:** Antibiotic consumption in DDD/100 bed-days for each antibiotic in the study wards, ACSH

Antibiotics	ATC code	Group of antibiotics	Route of administration	DDDs/100 bed-days
Gentamycin	J01GB03	Aminoglycoside	Injection	0.3
Ampicillin	J01CA01	Penicillin	Injection	1.3
Doxycycline	J01AA02	Tetracycline	Oral	2.9
Cefalexin	J01DB01	1st -generation cephalosporin	Oral	0.8
Metronidazole	J01XD01	Imidazole derivatives	Injection	45.5
Metronidazole	P01AB01	Imidazole derivatives	Oral	0.2
Cefazolin	J01DB04	1st -generation cephalosporin	Injection	1.4
Amoxicillin-clavulanic acid	J01CR02	Penicillin + beta-lactamase inhibitors	Oral	0.4
Trimethoprim-sulfamethoxazole	J01EE01	Sulfonamides and trimethoprim	Oral	0.2
Amoxicillin	J01CA04	Penicillin	Oral	0.4
Ceftriaxone	J01DD04	3rd -generation cephalosporin	Injection	73.2
Azithromycin	J01FA10	Macrolides	Oral	8.2
Vancomycin	J01XA01	Glycopeptide	Injection	19.5
Ciprofloxacin	J01MA02	Floroquinolone	Oral	3.2
Ciprofloxacin	J01MA02	Floroquinolone	Injection	0.5
Cefepime	J01DE01	4th -generation cephalosporin	Injection	0.6
Ceftazidime	J01DD02	3rd -generation cephalosporin	Injection	18.0
Cefixime	J01DD08	3th -generation cephalosporin	Oral	0.07
Meropenem	J01DH02	Carbapenem	Injection	3.4

**Fig. 3:**
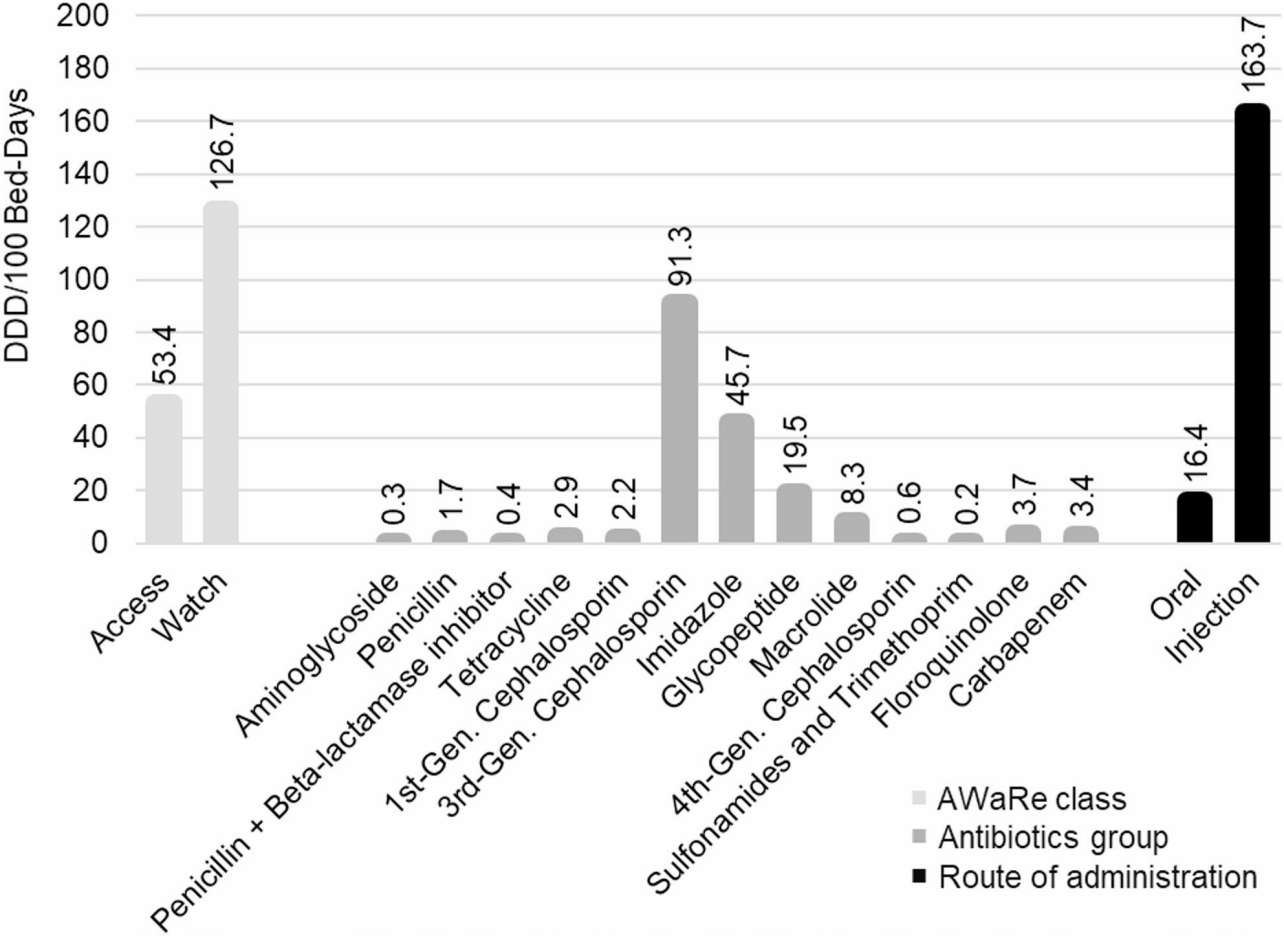
DDD/100 bed-days based on the WHO-AWaRe classification, antibiotic group and route of administration in the study wards, ACSH

The analysis revealed that the injection antibiotics (163.7 DDD/100 bed-days) resulted in greater consumption than the oral antibiotics (16.4 DDD/100 bed-days). In terms of the WHO-AWaRe class of antibiotic consumption, Access antibiotics accounted for 53.4 DDD/100 bed-days, whereas Watch antibiotics accounted for 126.7 DDD/100 bed-days. Furthermore, in terms of group of antibiotics, the 3rd generation cephalosporin group of antibiotics presented the highest DDD/100 bed-days (91.3), followed by the imidazole (45.7 DDD/100 bed-days) and glycopeptide (19.5 DDD/100 bed-days) groups of antibiotics (Fig. 3).

With respect to the calculated DDDs/100 bed-days of the wards, the medical ward had the maximum value (83.5 DDD/100 bed-days), and the Gyn/Obs ward had the minimum value (21.4 DDD/100 bed-days). The 3rd generation cephalosporin group of antibiotics had high consumption in the surgical (42.9 DDD/100 bed-days), medical (39.4 DDD/100 bed-days), and Gyn/Obs wards (8.9 DDD/100 bed-days), followed by the imidazole group of antibiotics (Table 4).

**Table 4:** Antibiotic consumption in DDD/100 bed-days in the study wards, ACSH

Antibiotic Group	ATC Code	Wards, DDD/100 bed-days			Total DDD/100 bed-days
		Surgical	Medical	Gyn/Obs	
Aminoglycoside	J01GB	0	0.3	0	0.3
Penicillin	J01CA	0	1.7	0	1.7
Tetracycline	J01AA	0	0.7	2.2	2.9
1st-gen. Cephalosporin	J01DB	0.1	0.6	1.5	2.2
3rd-gen. Cephalosporin	J01DD	42.9	39.4	9.0	91.3
4th-Gen. Cephalosporin	J01DE	0	0	0.07	0.07
Imidazole Derivative	J01XD; P01AB	25.9	12.0	7.8	45.7
Penicillin + Beta-lactamase inhibitor	J01CR	0	0.4	0	0.4
Sulfonamides and Trimethoprim	J01EE	0	0.2	0	0.2
Macrolide	J01FA	0	7.9	0.3	8.2
Glycopeptide	J01XA	4.2	14.8	0.5	19.5
Floroquinolone	J01MA	0.3	3.3	0	3.6
Carbapenem	J01DH	1.6	1.5	0.3	3.4
**Total**,** DDD/100 bed-days**		75.0	83.5	21.4	180.1

**Fig. 4:**
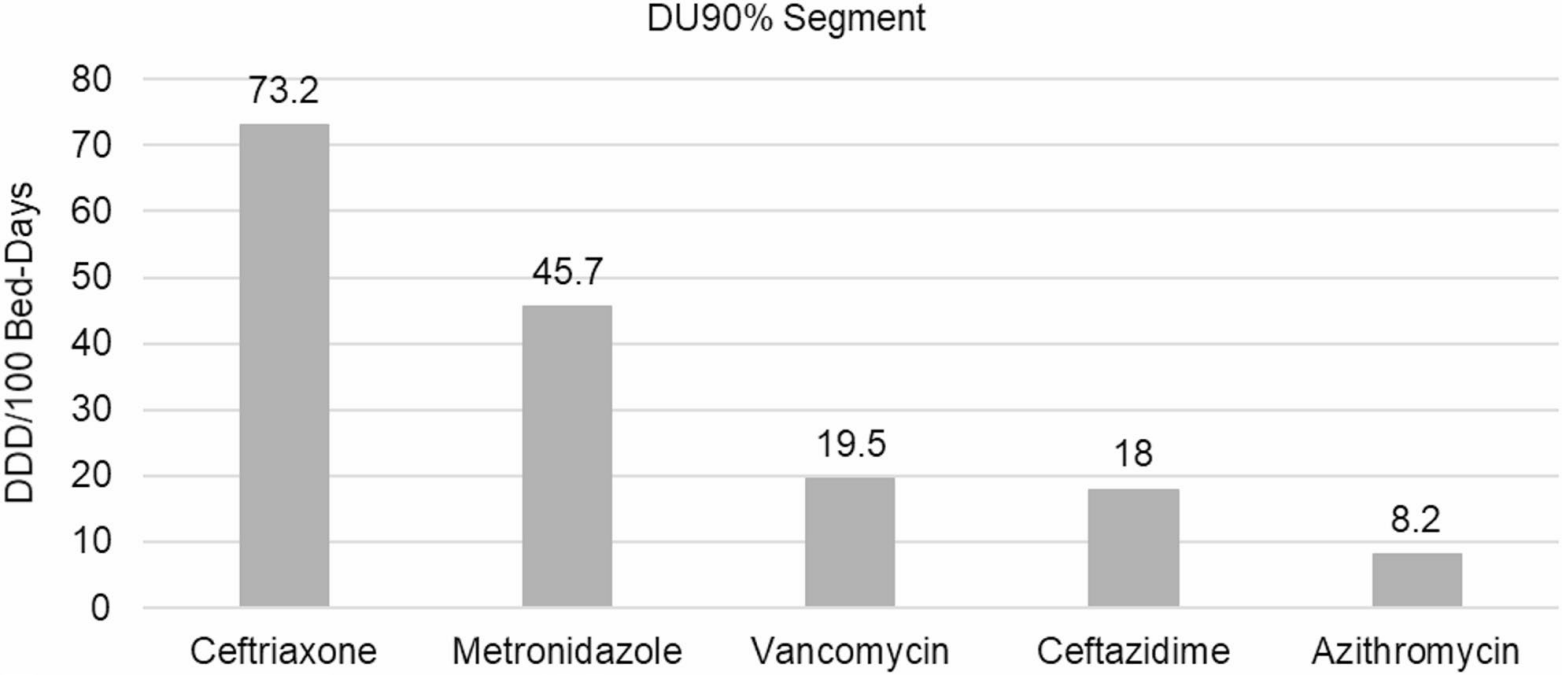
DU90% antibiotic consumption pattern in the study wards, ACSH

The DU90% segment in this study consisted of five antibiotics, four Watch and one Access class of antibiotics, accounting for 66% and 25.4% of the total consumption, respectively. The five antibiotics used were ceftriaxone, metronidazole, vancomycin, ceftazidime, and azithromycin, which together accounted for 91.4% of the total antibiotic consumption in DDD/100 bed-days (Fig. 4). Ceftriaxone alone contributed the largest share, representing 40.6% of the total consumption, followed by metronidazole (25.4%) and vancomycin (10.8%).

## Discussion

This study evaluated inpatient antibiotic prescribing patterns and consumption at a major comprehensive specialized tertiary hospital in northern Ethiopia using WHO-recommended methodologies. The findings demonstrate a clear predominance of Watch-category antibiotics, high overall antibiotic consumption, and substantial reliance on broad-spectrum agents, particularly ceftriaxone, underscoring important antimicrobial stewardship concerns in a referral-level setting.

According to the WHO-AWaRe classification, Watch antibiotics accounted for 64.1% of all prescribed antibiotics, while Access antibiotics constituted only 35.9%. This pattern deviates from the recommended level that at least 70% of antibiotic use should originate from the Access group [9]. Similar dominance of Watch antibiotics has been reported in other low- and middle-income countries (LMICs) hospital settings, including Zambia (43.1%) [33], Pakistan (45%) [17], India (53.2%) [16], Tanzania (37.4%) [18] and Sierra Leone (71.7%) [22], where tertiary and teaching hospitals frequently report higher proportions of Watch antibiotics due to empiric treatment norms and diagnostic limitations, as it is also highlighted in a recent multi-country review of AWaRe surveillance studies in LMICs [33]. Other possible contributors maybe related to concerns about treatment failure with Access antibiotics, and the availability and prescriber familiarity with Watch antibiotics. Similarly, Gandra and Kotwani, in a multi-centre study from India using the WHO AWaRe framework, reported higher use of Watch group antibiotics, which they attributed to concerns about treatment failure with Access antibiotics and the greater availability of Watch antibiotics [34]. In the Ethiopian context, high resistance to commonly used Access antibiotics has been well documented. Uropathogenic *E. coli* shows resistance rates of approximately 85% to ampicillin and 83% to amoxicillin, with cotrimoxazole resistance exceeding 57%, while *Staphylococcus aureus* exhibits similarly high resistance to ampicillin and amoxicillin in pooled analyses [35, 36]. Such resistance patterns may contribute to prescribers’ preference for broader-spectrum Watch antibiotics in empirical therapy.

The overall antibiotic consumption in this study (180.1 DDD/100 bed-days) was higher than that reported in several LMIC hospital-based studies, including those from Turkey (63.1 and 93.6) [21], Lebanon (72.6) [37], Sierra Leone (66.9) [22], Palestine (107.9) [24] and two hospitals in Eritrea (75.5 and 158.5) [38]. This elevated consumption may partly reflect the role of the study hospital as a major referral centre managing complex and severe cases, which often uses empirical and prolonged antibiotic therapy. Nevertheless, when ward-level consumption was examined, values were comparable to those reported in similar tertiary-care settings, such as Addis Ababa, Ethiopia [19] which reported values of 91.8, 47.6 and 71.6 for medical, Gyn/Obs and surgical wards, respectively. This study reported highest antibiotic consumption in the medical ward (83.5), followed by the surgical ward (75.0) and the Gyn/Obs ward (24.1). Evidence from comparable tertiary-care hospitals suggests that elevated consumption is closely linked to prolonged hospital stays, severity of illness, and extended empirical treatment courses [19, 39, 40].

Ceftriaxone was the most consumed antibiotic, followed by metronidazole and vancomycin. This pattern is consistent with findings from other Ethiopian [19] and LMIC studies [20, 22, 41, 42], where ceftriaxone is frequently preferred because of its broad-spectrum activity, and perceived effectiveness in empirical therapy and surgical prophylaxis. Despite ceftriaxone being the highest consumed antibiotic in our study, local antimicrobial resistance data indicate that its effectiveness may be compromised for many pathogens in Ethiopia. National systematic reviews show ceftriaxone resistance rates of 38–74% among common gram-negative bacteria such as *E. coli* and Klebsiella species [43]. Additionally, studies from Ethiopian referral hospitals have reported resistance as high as 78–96% for Enterobacteriaceae [44], and a hospital study found a 57.2% overall ceftriaxone resistance rate among clinical isolates [45], while the national pooled prevalence of inappropriate ceftriaxone utilisation was 55.2% [46].

Analysis by antibiotic class further demonstrated that third-generation cephalosporins (91.3 DDD/100 bed-days) accounted for the highest consumption across all wards, reinforcing concerns about inappropriate spectrum selection. Similar trends have been reported in LMIC hospital settings, such as Ethiopia [19] and Turkey [20], where limited access to microbiological diagnostics and stewardship oversight often drive empirical use of broad-spectrum antibiotics. The excessive use of 3rd -generation cephalosporins is very concerning, as it predisposes to the development of extended-spectrum beta-lactamase (ESBL)-producing microorganisms [47].

The DU90% analysis showed that five antibiotics accounted for the majority of consumption, with Watch antibiotics comprising two-thirds of the DU90% segment. This figure was lower than that reported in studies conducted in Ethiopia (6–8 antibiotics) [19] and India (5–13 antibiotics) [48] but higher than that reported in studies conducted in Palestine (4 antibiotics) [24]. While a limited DU90% list may indicate prescribing concentration, dominance by Watch antibiotics reflects suboptimal alignment with stewardship principles and highlights opportunities for targeted interventions.

Evidence demonstrates that structured antimicrobial stewardship programmes are effective in reducing unnecessary antibiotic use, improving prescribing appropriateness, and impacting resistance trends in hospital settings. A large systematic review and meta-analysis found that implementation of antimicrobial stewardship programmes was associated with about a 28% reduction in overall antibiotic use and specifically reduced consumption of WHO Watch antibiotics across diverse healthcare settings, particularly when guideline development, audit and feedback, and prescriber education were combined [49]. In LMICs, antimicrobial stewardship programmes implementation has been shown to improve the rational use of antibiotics despite contextual barriers such as limited diagnostics and guideline infrastructure [50]. A recent scoping review reported reduced antibiotic consumption and improved prescribing with stewardship interventions in LMIC hospitals, although resources and human capacity remain challenges [30]. Administration of antibiotics by an anaesthesia technician instead of the ward nurse for the initial prophylactic antibiotic dose for one year as an intervention resulted in a reduced antibiotic consumption from 93.6 to 63.1 DDD/100 bed-days [21]. These findings show that the introduction of robust or model clinical guidelines for therapeutic and prophylactic purposes can greatly reduce the consumption of antibiotics and help slow the occurrence of AMR.

This study found out major antimicrobial stewardship gaps in a tertiary-care hospital, reflected by the dominance of Watch antibiotics, high overall consumption, and heavy reliance on ceftriaxone, which deviate from WHO AWaRe recommendations. Though, the hospital had an antimicrobial stewardship committee at the time of the study, its functionality was limited. Prescribing was largely clinician-driven, with minimal guideline use, audit, or multidisciplinary oversight, and highly restricted access to microbiology testing further constrained evidence-based decisions. These findings provide baseline evidence to inform strengthening of antimicrobial stewardship practices. Strengthening the hospital stewardship programs, improving access to microbiological diagnostics, and institutionalizing routine monitoring using AWaRe and DDD metrics are urgently needed. The results also provide baseline data for evaluating future stewardship interventions.

## Limitations of the study

To the best of our knowledge, this is the first study to evaluate antibiotic prescribing patterns via the WHO-AWaRe classification and consumption pattern using ATC/DDD and DU90% methodologies in the study area. This study also used a large sample size. However, certain limitations should be considered when interpreting the results. This prospective cross-sectional study was conducted exclusively at ACSH, without parallel studies in other local hospitals, limiting the ability to compare antibiotic prescribing consumption patterns with other facilities in the region. Although the use of quota sampling was employed to ensure proportional representation of major inpatient wards and to maintain feasibility in a high-volume tertiary-care hospital, it may introduce selection bias. The study focused only on inpatients, so the findings do not represent outpatient settings. Additionally, this study did not include intensive care units, where antibiotics are more frequently used; therefore, this may have led to an underestimation of antibiotic use. Furthermore, antibiotic consumption analysis using DDD/100 bed-days was restricted to adult inpatients due to the lack of validated DDDs for paediatric patients [10].

## Conclusion

This study revealed that antibiotic prescribing at Ayder Comprehensive Specialized Hospital is heavily dominated by Watch antibiotics, particularly ceftriaxone, whereas Access antibiotics were well below the recommended threshold of 70%. Overall antibiotic consumption, expressed in DDD/100 bed-days, was high compared with reports from other countries. The DU90% analysis further confirmed the concentrated use of a small group of broad-spectrum antibiotics. These patterns highlight irrational prescribing practices, overreliance on third-generation cephalosporins, and limited alignment with global stewardship targets. Strengthening antimicrobial stewardship interventions, enforcing evidence-based prescribing guidelines, such as clinical guidelines for infectious diseases, and promoting culture and sensitivity-guided therapy are urgently needed to optimize antibiotic use and curb the threat of AMR in Ethiopia.

## Supplementary Information

Below is the link to the electronic supplementary material.

Supplementary Material 1

## Data Availability

The data used and/or analysed during the study are available from the corresponding author upon reasonable request.

## Abbreviations

ACSH: Ayder Comprehensive Specialized Hospital
AWaRe: Access, Watch, and Reserve
ATC: Anatomical Therapeutic Chemical
AMR: Antimicrobial Resistance
DDD: Defined Daily Dose
Gyn/Obs: Gynecology/obstetrics
LMIC: Low- and Middle-Income Countries
WHO: World Health Organization
