# Supplementary material for: Antibiotic prescribing patterns and consumption at a comprehensive specialized hospital in northern Ethiopia: a prospective cross-sectional study underscoring the need for antimicrobial stewardship
Source: BMC Infect Dis. 2026 Apr 4;26:962. doi: 10.1186/s12879-026-13254-1 (PMC13182102; doi:10.1186/s12879-026-13254-1)
Supplement: Supplementary file 1 — Supplementary Material 1 [file 12879_2026_13254_MOESM1_ESM.docx]

**Appendix A. Data Abstraction Format for Antibiotic Prescribing and Consumption Assessment at ACSH**

1. **Antibiotic Prescribing Pattern**

Name of Unit/Ward: _____________ Data collector: _______________________________________ Date: ___________________

| **MRN** | **Diagnosis** | **Age** | **Sex** | **Residence** | **Admission Date** | **Discharge Date** | **Purpose of therapy** | **Comorbidity** | **No. of Days in Hospital** | **Culture?** | **Sensitivity**  **Test Done?** | **Name(s) of Antibiotic(s) Prescribed** | **AWaRw class** | **Dosage Form and Strength** | **Total Days of Treatment** | **Dosage**  **Frequency**  **per Day** | **Doses**  **Administered** |
| --- | --- | --- | --- | --- | --- | --- | --- | --- | --- | --- | --- | --- | --- | --- | --- | --- | --- |
|  |  |  |  |  |  |  |  |  |  |  |  |  |  |  |  |  |  |
|  |  |  |  |  |  |  |  |  |  |  |  |  |  |  |  |  |  |
|  |  |  |  |  |  |  |  |  |  |  |  |  |  |  |  |  |  |
|  |  |  |  |  |  |  |  |  |  |  |  |  |  |  |  |  |  |
|  |  |  |  |  |  |  |  |  |  |  |  |  |  |  |  |  |  |
|  |  |  |  |  |  |  |  |  |  |  |  |  |  |  |  |  |  |
|  |  |  |  |  |  |  |  |  |  |  |  |  |  |  |  |  |  |

1. **Antibiotic Consumption Assessment**

| **MRN** | **Name of antibiotic** | **Ward name** | **Total Consumed Dose in g** | **ATC Code** | **WHO DDD in g** |
| --- | --- | --- | --- | --- | --- |
|  |  |  |  |  |  |
|  |  |  |  |  |  |
|  |  |  |  |  |  |
|  |  |  |  |  |  |
|  |  |  |  |  |  |
